# Cost-utility analysis of transcranial direct current stimulation therapy with and without virtual illusion for neuropathic pain for adults with spinal cord injury in Canada

**DOI:** 10.1080/10790268.2021.1961051

**Published:** 2021-11-15

**Authors:** Min Xi, XiaoWei Shen, Kamilla Guliyeva, Rebecca Hancock-Howard, Peter C. Coyte, Brian C. F. Chan

**Affiliations:** 1 KITE – Toronto Rehab Institute – University Health Network, Toronto, Ontario, Canada; 2 Institute of Health Policy, Management and Evaluation, University of Toronto, Toronto, Ontario, Canada; 3 Hoffmann-La Roche Limited, Toronto, Ontario, Canada

**Keywords:** Spinal cord injury, Neuropathic pain, Cost-utility, Virtual illusion, Transcranial direct current stimulation

## Abstract

**Objective:**

To undertake a cost-utility analysis comparing virtual illusion (VI) and transcranial direct current stimulation (tDCS) combination therapy, tDCS alone and standard pharmacological care in Ontario, Canada from a societal perspective over a three-month time horizon.

**Design:**

Cost-utility analysis using Markov model methods

**Setting:**

Community setting in Ontario, Canada.

**Participants:**

Individuals with spinal cord injury and neuropathic pain (NP) resistant to pharmacological therapy.

**Interventions:**

Virtual illusion and transcranial direct current stimulation, transcranial direct current stimulation alone and standard pharmacological therapy.

**Outcome Measures:**

Incremental costs, quality adjusted life years (QALY) and incremental cost effectiveness ratio

**Results:**

The incremental cost effectiveness ratio of VI and tDCS therapy cost is $3,396 per QALY (2020 Canadian dollars) when compared to standard care. The incremental cost per QALY of tDCS therapy alone is $33,167. VI and tDCS therapy had lower incremental costs (−$519) and higher incremental QALYs (0.026) compared to tDCS alone. From a public healthcare payer perspective, there is a 74% probability that VI and tDCS therapy and 54% probability that tDCS alone would be cost effective at a $50,000 per QALY willingness-to-pay threshold. Our findings remained relatively robust in various scenario analyses.

**Conclusion:**

Our findings suggest that at three-months after therapy, VI and tDCS combination therapy may be more cost effective than tDCS therapy alone. Based on conventional health technology funding thresholds, VI and tDCS combination therapy merits consideration for the treatment of NP in adults with spinal cord injuries.

## Background

In Canada, there are over 85,000 individuals living with spinal cord injury (SCI) in the community.^1^ Eighty percent of these cases experience some level of neuropathic pain (NP).^2^ NP is defined by the International Association of the Study of Pain (IASP) as “pain caused by a lesion or disease of the somatosensory nervous system”.^3^

The most common treatment for NP are pharmacological, with gabapentinoids, tricyclic antidepressants and serotonin–norepinephrine reuptake inhibitors recommended as first line treatments.^4,5^ However, most individuals receiving these treatments do not achieve clinically significant pain reduction long-term which may have a large impact on the health-related quality of life and costs associated with NP.^6^ For example, individuals receiving pharmacological treatments to reduce NP severity may experience adverse events including difficulty concentrating, drowsiness, lack of appetite, dizziness, and/or issues urinating.^7^ These adverse events may lead to poor health-related quality of life, decreased productivity, and a high frequency of healthcare utilization, which may result in with high healthcare and societal costs associated with NP despite pharmacological therapy.^8–10^

As a result, alternative non-pharmacological interventions may be considered for adjuvant therapy. Transcranial direct current stimulation (tDCS) is a non-invasive brain stimulation therapy used to treat various conditions including NP. tDCS involves the non-invasive transfer of a low intensity electrical current to an individual’s head, typically delivered via two large electrodes, operated by a small battery-powered device.^11^ Placement of electrodes are intended to regulate current flow to specific regions of the brain associated with pain relief. In most studies, the duration of tDCS sessions was 20 min, with an intensity ranging from 1–2 mA, and repeated daily from between five to 10 sessions.^12^ A recent systematic review pooled NP treatment effect from clinical studies using standardized mean differences because of the heterogeneity in pain intensity, depression and anxiety assessment scales.^13^ The synthesis of the data observed no statistically significant reduction in pain reduction. However, the power of the pooled pain reduction estimates was limited by the small number of included studies and the small sample sizes of these studies. Additionally, the studies included in the review involved predominantly male participants so the pooled pain reduction estimates may not be generalizable across gender. Current clinical evidence has observed that the inclusion of Virtual Illusion (VI) to tDCS has shown improved outcomes compared to tDCS alone.^14^ VI therapy is a non-immersive virtual reality therapy that can be used to reduce NP in individuals living with SCI.^14^ Non-immersive virtual reality does not require head mount equipment, allowing participants to maintain connection to the real world environment.^14^ VI therapy involves the use of cognitive techniques such as guided images and videos to modify behaviors and alter tactical perceptions.^15–17^ VI therapy for individuals with SCI involves individuals sitting approximately 2.5 m in front of a screen that projects a video of a person walking.^15–17^ A vertical mirror is placed in front of the individual on top of the screen to induce greater gait perception realism, due to the alignment of the patient’s own upper body and the video of walking legs projected onto the screen.^15–17^ When used in conjunction with tDCS at an intensity ranging from 1-2mA, VI is usually initiated five minutes following the start of tDCS and lasts for 15 min, repeated daily for 10 sessions.

Studies have demonstrated that the simultaneous administration of tDCS and VI therapy, rather than tDCS alone, offers several advantages including a greater, significant, and maintained reduction in pain intensity perception over a 12 week period.^15,16^ However, both VI therapy and tDCS comes at additional equipment and personnel costs.^18,19^ Previous studies^20,21^ have shown that virtual reality rehabilitation therapies involving the use of motion-sensing devices for 45–50 min sessions three days per week for seven to 12 weeks may lead to cost savings in other populations. To date, no economic evaluations have been conducted for virtual reality therapies for NP in the SCI population. Similarly, there is a lack of evidence on the cost-effectiveness of tDCS in the SCI population. Thus, this study aims to investigate the cost-utility of in-hospital VI and tDCS combination therapy and tDCS alone compared to standard care for Canadian adults with SCI experiencing NP from the perspective of the public health-care payer and the individual receiving treatment. A cost-utility analysis will provide valuable information for public payers in determining whether the implementation of tDCS and VI therapy or tDCS alone will be good value for money.

## Methods

### Overview

A cost-utility analysis was used to determine the cost, quality-adjusted life years (QALY) gained, and incremental cost-utility ratio (ICER) of the tDCS and VI therapy and tDCS alone versus and standard care.^22^ This evaluation technique was chosen since QALY is a generic health outcome measure, allowing for broad comparisons across different health conditions and interventions. Standard care consists of first-line pharmaceutical intervention for NP. The analysis was carried out from a Canadian societal perspective where all interventions costs irrespective of payer are included in the analysis.^23^ Specifically, this perspective includes all health care expenditures accrued by residents in Ontario paid for by the Ministry of Health and Long Term Care (MoHLTC). The MoHLTC covers approximately 70% of Ontario’s healthcare expenses,^24^ providing a comprehensive picture of healthcare costs. The societal perspective incorporates the public health care payer expenditures and also includes the productivity costs due to time lost from work and non-work activities at each level of pain severity in the health states assessed.

### Model structure

**Figure 1:**
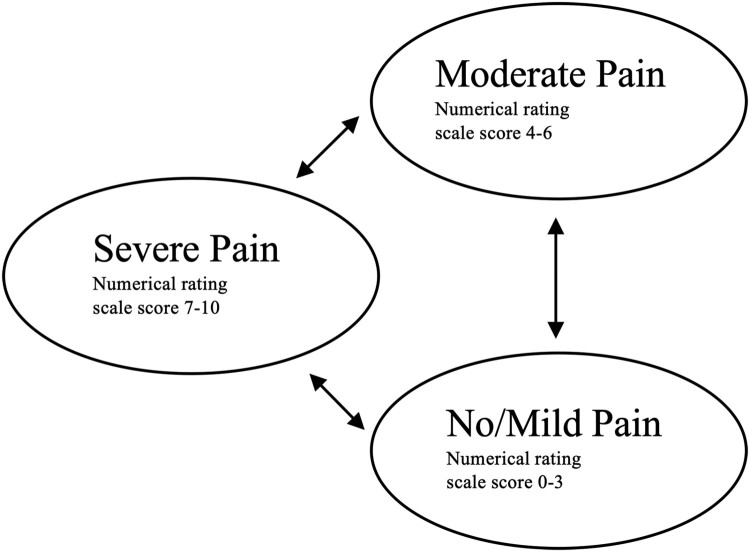
Markov model describing patient transition between pain states over a three-month time horizon.

A cohort of 1000 individuals with chronic SCI experiencing NP was simulated using a probabilistic Markov model. The model structure is similar to a recent economic evaluation of a pharmacological treatment for individuals with chronic cervical NP.^25^ The simulated model cohort could move between the three mutually exclusive health states according to NP severity. Severity was stratified using the numerical rating scale (NRS), the recommended measure for pain intensity following SCI.^26,27^ As in previous NP models,^28,29^ No/mild pain ranged from NRS 0–3, moderate pain ranged from NRS 4–6, and severe pain ranged from NRS 7–10.^30^ All individuals started in the severe pain health state, comparable to the baseline pain intensity of participants involved in tDCS and VI therapy^15,16^ and tDCS clinical trials.^31,32^ The cycle length was two weeks, after which an individual could transition from one health state to another or remain in the same health state. This length was chosen to match the follow-up period of the primary study outcomes incorporated into the model.^15^ A pictorial of the model is presented in Fig. 1. The model was developed using the “heemod” package^33^ in R 3.6.2.^34^

The model time frame for the primary analysis was three months in duration to match the follow-up period of the clinical trial by Soler and colleagues^15^ and similar to previous economic evaluations for NP.^25,29^ Since the time horizon was three months, mortality was not included in the model.

#### Cohort characteristics

Cohort characteristics were based on participant level demographic data from the primary clinical studies that inform the model inputs.^16,31^ Individuals in the model were adults over 18 years of age, with an SCI and severe NP (8 on the 11-point NRS) resistant to pharmaceuticals. Average age of the simulated model cohort was 46 ± 8.5 years, 66% male with an average time since SCI of 68 ± 37.7 months.

#### Data input

A brief literature search was conducted in PubMed and Google Scholar using search terms: virtual illusion, virtual walking, augmented reality, rehabilitation, transcranial direct current stimulation, spinal cord injury, neuropathic pain, pain, chronic pain, cost, cost-effectiveness, and cost-utility. Studies focussing on NP and SCI, studies written in English, and studies conducted in Canada were prioritized as sources of data input.

Data from four clinical trials^15,16,31,32^ were used to estimate transition probabilities following treatment with tDCS alone and tDCS and VI therapy. The transition probability for the initial two-week cycle was derived from pain data before and following a two-week period of tDCS and VI or tDCS alone.^16,31^ Transition probabilities for subsequent two-week cycles were derived from observations of pain scores at subsequent two-week follow-ups.^15,32^ For tDCS and VI treatment, transition probabilities were based on observations of sustained outcomes at three months.^15^ For tDCS alone, transition probabilities were derived from four-week follow-up data.^32^ It is assumed that these probabilities are also representative at two weeks. Transition probability model inputs are presented in Table 1.

**Table 1:** Transition probability and utility (2020 Canadian dollars) model inputs.

Parameter	Model inputs	Distribution type	Reference
**Transition probabilities for transcranial direct current stimulation and virtual illusion cohort, mean (standard deviation)**			
**Cycle 0**			
Severe to mild	0.19 (0.1)	Multinomial	Kumru *et al.*^16^
Severe to moderate	0.63 (0.1)	Multinomial	
Severe to severe	0.19 (0.1)	Multinomial	
**Cycles 1–6**			
Mild to mild	0.95 (0.1)	Multinomial	Soler *et al.*^15^
Mild to moderate	0.05 (0.1)	Multinomial	
Mild to severe	0 (0.1)	Multinomial	
Moderate to mild	0.05 (0.1)	Multinomial	
Moderate to moderate	0.95 (0.1)	Multinomial	
Moderate to severe	0 (0.1)	Multinomial	
Severe to mild	0 (0.1)	Multinomial	
Severe to moderate	0.05 (0.1)	Multinomial	
Severe to severe	0.95 (0.1)	Multinomial	
**Transition probabilities for transcranial direct current stimulation cohort, mean (standard deviation)**			
**Cycle 0**			
Severe to mild	0.1 (0.1)	Multinomial	Yoon *et al.*^31^
Severe to moderate	0.5 (0.1)	Multinomial	
Severe to severe	0.4 (0.1)	Multinomial	
**Cycles 1–6**			
Mild to mild	0.5 (0.1)	Multinomial	Wrigley *et al.*^32^
Mild to moderate	0.5 (0.1)	Multinomial	
Mild to severe	0 (0.1)	Multinomial	
Moderate to mild	0 (0.1)	Multinomial	
Moderate to moderate	0.4 (0.1)	Multinomial	
Moderate to severe	0.6 (0.1)	Multinomial	
Severe to mild	0 (0.1)	Multinomial	
Severe to moderate	0.33 (0.1)	Multinomial	
Severe to severe	0.67 (0.1)	Multinomial	
**Health state utilities, mean (standard deviation)**			
Mild	0.77 (0.1)	Beta	Gu *et al.*^49^
Moderate	0.63 (0.1)	Beta	
Severe	0.44 (0.1)	Beta	

### Cost data

All source cost data were inflated to 2020 Canadian dollars using the Bank of Canada Cost Inflation Calculator.^35^ The initial cost of the intervention and comparator were calculated using information received from a manufacturer of tDCS equipment (neuroCare Group GmbH), market costs of VI equipment^36–41^ and the Ontario Schedule of Benefits^42,43^ and Canadian guidelines for personnel costs.^44,45^ Intervention cost model inputs are presented in Table 2. Additional per cycle costs were calculated based on the resource utilization associated with each health state. Resource utilization estimates for individuals with NP and SCI, including probability of a physician visit, probability of a specialist referral, probability of diagnostic test ordered referred to a specialist, and drug costs, were obtained from a previous economic evaluation of pregabalin for diabetic peripheral neuropathy and postherpetic neuralgia NP.^29^ The probability of a physician visit, probability of a specialist referral, and cost of drugs ordered were dependent on the individual’s health state and the probability of a diagnostic test ordered was dependent on the probability of a physician visit and specialist referral.^29^ Physician and specialist costs in addition to costs of diagnostic tests were weighed according to the probability of healthcare utilization of each healthcare service.^29^ Detailed healthcare probabilities and costs are presented in Appendix Table A1.

**Table 2:** Transcranial direct current stimulation and virtual illusion intervention cost (2020 Canadian dollars) model inputs.

Parameter	Model inputs	Distribution type	Reference
**Cost of transcranial direct current stimulation per patient for 20 sessions, mean (standard deviation)**			
tDCS equipment (current stimulator, anodes)^a^	70 (5)	Gamma	Costs from manufacturer
Physician	1,228	–	Ontario Physician Schedule of Benefits (2020; A003 × 20)^42^
Nurse^b^	370.23	–	Ontario Nurses Association ($36.12/h × 10.25 h; 2020)^45^
Drugs	91.83 (95)	Gamma	Tarride *et al.*^46^
**Cost of virtual illusion per patient for 20 sessions, mean (standard deviation)**			
Projector^a^	8 (2)	Gamma	CostHelper Inc.^36^
Screen^a^	1.90 (1)	Gamma	Staples Canada ULC^38^
Video^a^	30 (8)	Gamma	Base Two Media Inc.^37^
Portable computer^a^	9.67 (3)	Gamma	Statistica^39^
Vertical mirror^a^	1.8 (1)	Gamma	Fixr^40^
Loudspeakers^a^	2.5 (1)	Gamma	CostHelper Inc.^41^
Physiotherapist^b^	922.71	–	Financial Fee Services Ontario^44^

^a^ All equipment costs were calculated assuming a five year lifespan as per Sauvaget *et al.*^19^.

^b^ Personnel costs accounted for registration, equipment set up and take down time. First session was 45 min and subsequent sessions were 30 min as per Sauvaget *et al.*^19^.

Productivity costs were calculated using the human capital method.^46^ The amount of time lost per month from both paid work and unpaid work (e.g. daily activities, volunteer work) due to NP per month were obtained from a survey of Canadians suffering from NP and SCI.^46^ The hours lost from paid and unpaid work were divided by two to fit our two-week cycles. The cost of lost productivity due to time lost from paid work were calculated by multiplying the number of hours of paid work lost by the 2020 average hourly wage from Statistics Canada.^46,47^ Lost productivity costs due to time lost from unpaid work were calculated by multiplying the number of hours of unpaid work lost by the 2020 minimum hourly wage in Ontario.^48^ Productivity unit costs are presented in Appendix Table A2.

### Utility data

To determine the QALY of tDCS and VI therapy versus tDCS alone, we used health utilities derived by Gu and colleagues^49^ from 2,719 adult respondents with NP. This study used an ordinary least squares regression model to map respondent NRS scores to EQ-5D utility values.^49^ Utility model inputs are presented in Table 1. Utility values were weighed by the time spent in each health state to calculate QALY. Both costs and outcomes were not discounted given that the model length was one year.

### Scenario and sensitivity analyses

The primary study outcome is the incremental cost per QALY, tDCS and VI therapy compared to standard care, and tDCS alone compared to standard care. This was calculated by dividing the incremental cost by the incremental QALY. We evaluated the impact of alternative model inputs on the incremental cost, QALYs, and ICER by conducting various scenario sensitivity analyses. Scenario analyses included are described in Table 3. Long-term direct healthcare^50,51^ and societal costs^46,51^ were estimated between three to twelve months for each health state (*i.e.* mild, moderate, and severe NP severity) and assigned to the proportion of individuals in each health state at the end of three months. Similar to a previous economic evaluation for NP, it was assumed that individuals did not change NP severity health states from three months to one year.^25^ A two year time frame was also included in a separate scenario. Long-term costs are presented in Appendix Table A3. Public health-care payer perspective was examined separately as a scenario analysis. Uncertainty in the primary study outcome resulting from variability in the model inputs was assessed by conducting a probabilistic sensitivity analysis using a Monte Carlo simulation. More specifically, the study model was repeated 1,000 times. For each iteration, model inputs were extracted from random number pulls confined by a distribution assigned to each input. Cost inputs followed a gamma distribution, transition probabilities had multinomial distributions, utilities and probabilities of health care utilization were assigned a beta distribution as recommended by Briggs and colleagues.^52^ The details of model inputs and distributions are presented in Tables 1 and 2.

**Table 3:** Description of scenarios for sensitivity analysis.

Scenario	Description	References
**Perspective**		
Public healthcare payer	Productivity costs excluded from the analysis.	–
**Timeframe**		
1 year	The base-case model of 3 months changed to 1 year.	–
2 year	The base-case model of 3 months changed to 2 years. Applied a discount rate of 1.5% per annum as per CADTH guidelines.^23^	–
**Utilities**		
Alternative set of utilities 1	Utility for mild health state changed from 0.77 in base case to 0.93. Utility for moderate health state changed from 0.63 in base case to 0.8. Utility for severe health state changed from 0.44 in base case to 0.34.	Dixon *et al.*^27^
Alternative set of utilities 2	Utility for mild health state changed from 0.77 in base case to 0.71. Utility for moderate health state changed from 0.63 in base case to 0.47. Utility for severe health state changed from 0.44 in base case to 0.2.	Gordon *et al.*^49^
**Costs**		
Alternative VR equipment costs	Equipment costs changed to $3,500 per year.	Delshad *et al.*^55^

A cost-effectiveness acceptability curve is presented using the probabilistic sensitivity analysis results from the public healthcare payer perspective to evaluate the probability that tDCS and VI would be cost-effective for a willingness-to-pay thresholds ranging from 0 to $100,000 per QALY.

### Model assumptions

We assumed similar clinical management for the tDCS and VI group and the tDCS group. The pivotal clinical trial comparing tDCS and VI with tDCS alone did not observe differences in major adverse events between the two NP treatments arms during the 12-week follow-up period.^15^ It was assumed that this will continue to be the case at one year. Additionally, our model assumed no deaths within the time horizon applied. Finally, transition probabilities for cycles 1–6 were assumed to be the same, derived from studies with two and four week follow-up after treatment.^15,32^

## Results

**Figure 2:**
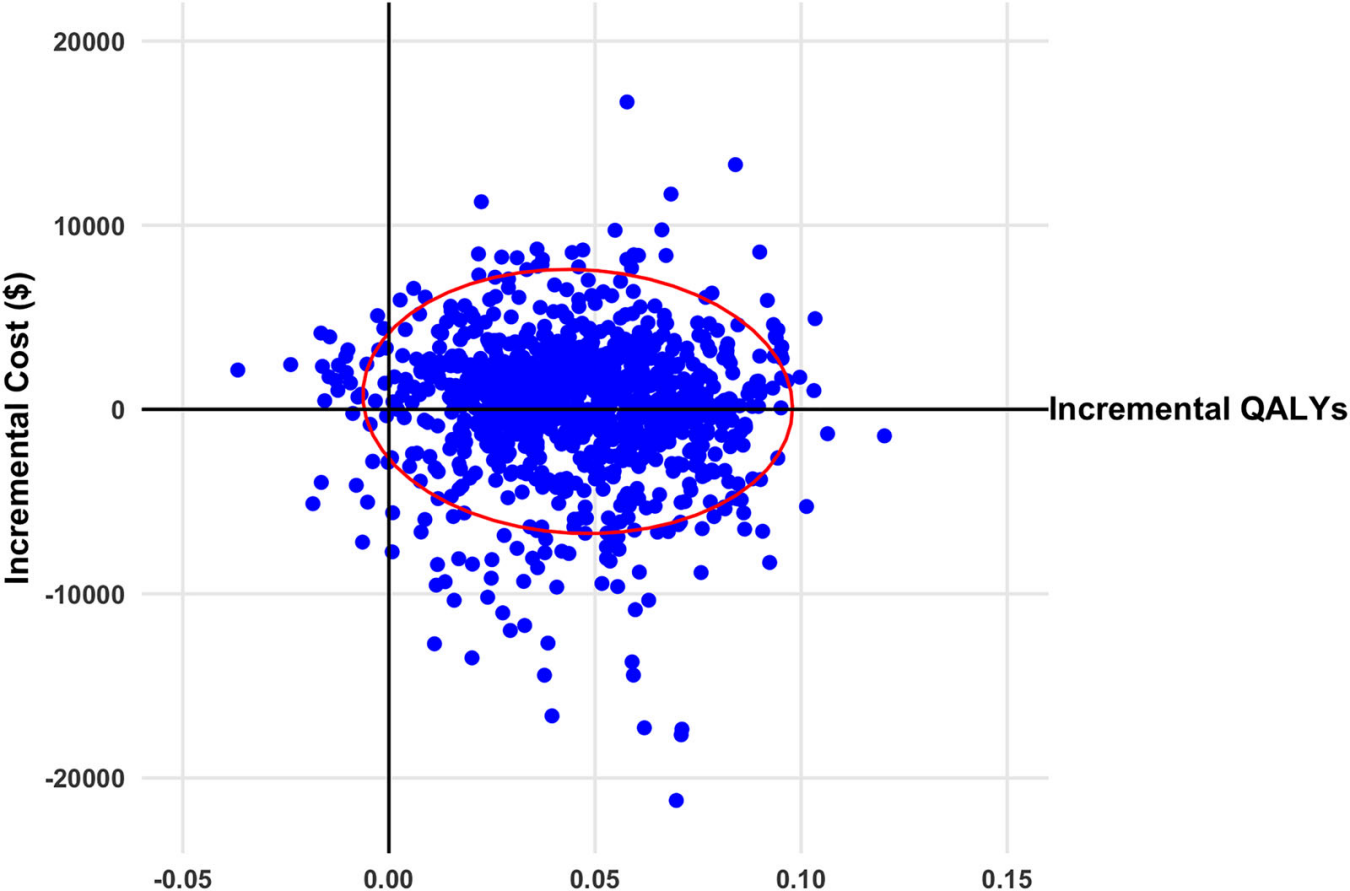
Scatterplot of incremental cost and quality adjusted life years (QALYs) for virtual illusion and transcranial direct current stimulation versus standard care from societal perspective (2020 Canadian dollars). Ellipsis represents 95% confidence interval.

**Figure 3:**
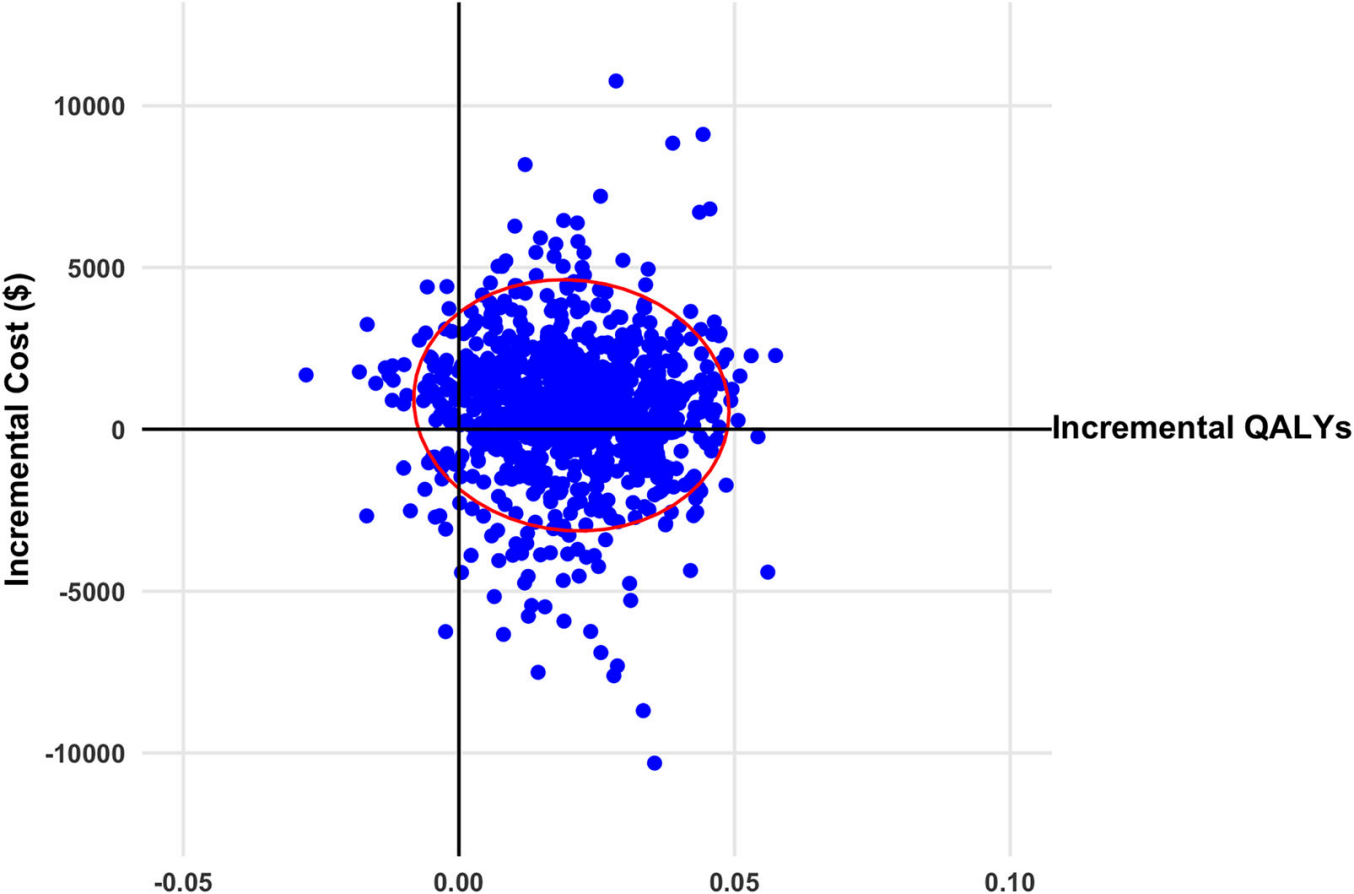
Scatterplot of incremental cost and quality adjusted life years (QALYs) for transcranial direct current stimulation versus standard care from societal perspective (2020 Canadian dollars). Ellipsis represents 95% confidence interval.

**Figure 4:**
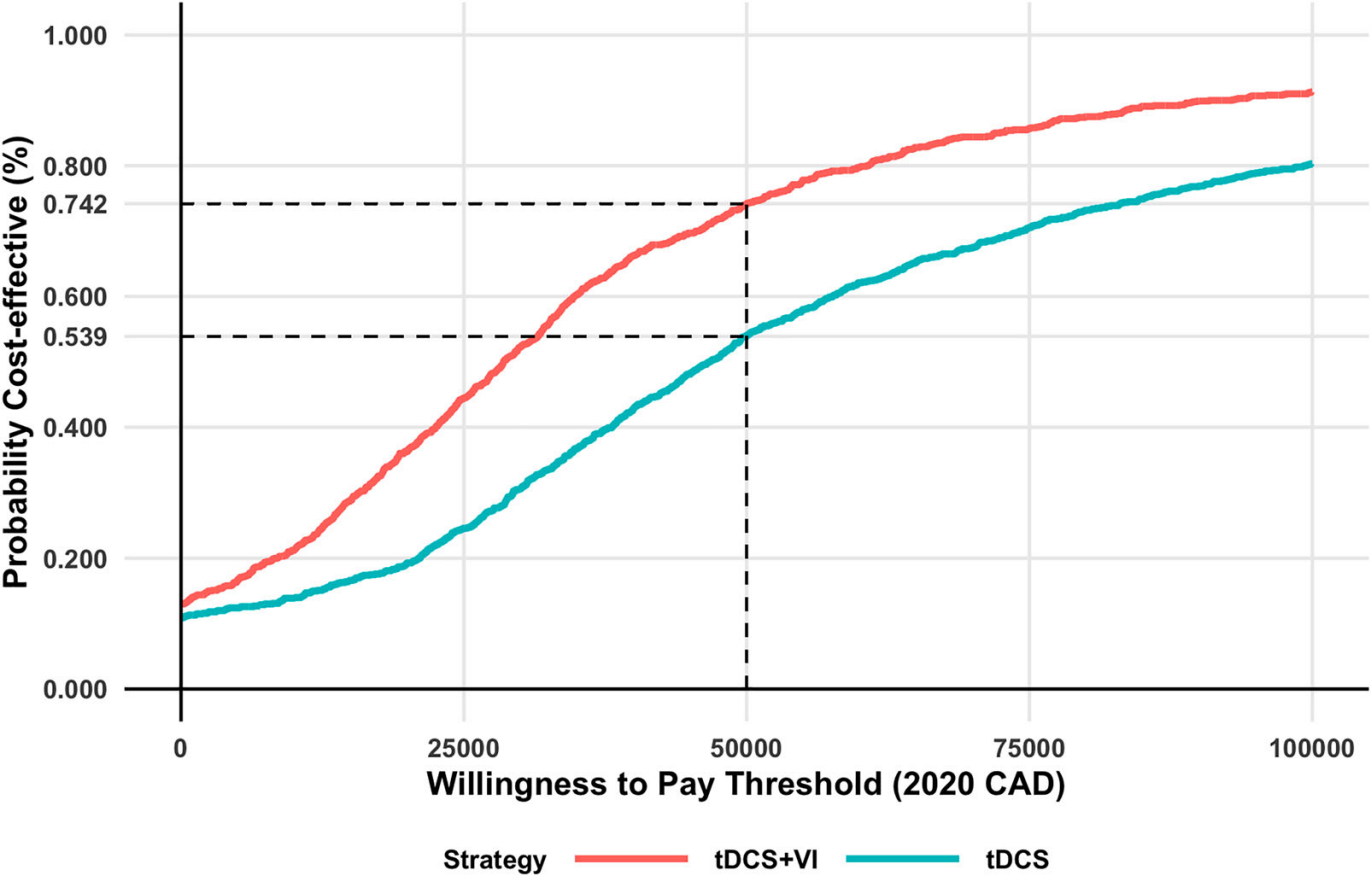
Cost-effectiveness acceptability curve for base case results from a public health care player perspective.

**Figure A1:**
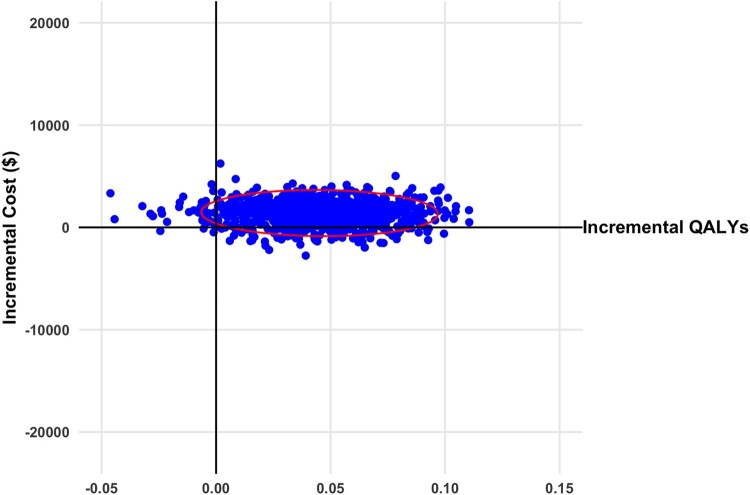
Scatterplot of incremental cost and quality-adjusted life year (QALYs) for virtual illusion and transcranial direct current stimulation versus standard care for the public health care payer perspective (2020 Canadian dollars).

**Figure A2:**
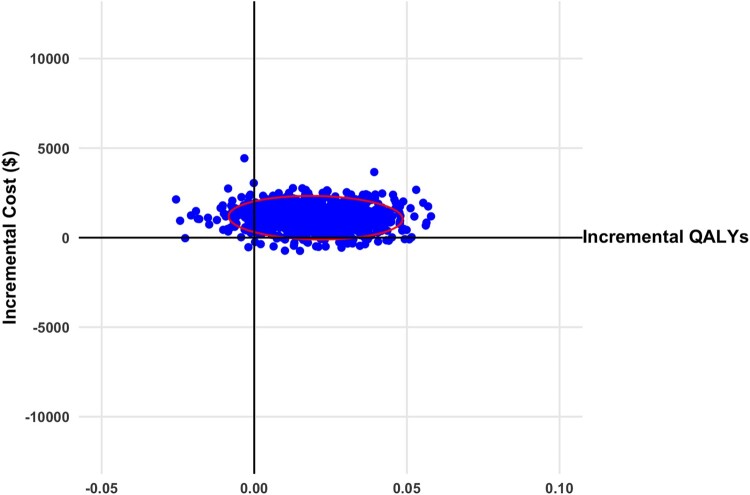
Scatterplot of incremental cost and quality-adjusted life years (QALYs) for transcranial direct current stimulation versus standard care base case analysis for the public health care payer perspective (2020 Canadian dollars).

At the three-month time point, the model projected that from a cohort receiving VI and tDCS therapy, 28%, 57%, and 15% would be in the mild, moderate, and severe health states, respectively. On the other hand, for a cohort receiving tDCS therapy, none were in the mild health state, 36% were in the moderate health state, and 64% were in the severe health state at three months. The base case total cost per person for the cohort receiving VI and tDCS is more than standard care. The incremental QALY for VI and tDCS was more than standard care. Similar observations were seen for tDCS alone. However, the incremental cost effectiveness ratio is almost ten times higher for tDCS compared to standard care. This is a result of a higher incremental cost and lower incremental QALYs of tDCS versus VI and tDCS when compared to standard care. The base case results are presented in detail in Table 4. VI and tDCS dominates when compared with tDCS alone, resulting in a lower cost of −$519 and an improved incremental QALYs of 0.026. The repeated model simulations comparing VI and tDCS and tDCS alone with standard care resulted in most ICERs with a positive incremental QALY and a mix of positive and negative incremental costs. The results from a societal perspective are presented on a cost-effectiveness plane in Figs. 2 and 3. The same results from a public healthcare payer perspective are presented in Appendix Figure A1 and A2. Examining the cost effectiveness acceptability curve from a public healthcare payer perspective, VI and tDCS had a 74% of being cost-effective at a willingness to pay (WTP) threshold of $50,000 per QALY gained and 90% at a WTP threshold of $100,000 per QALY (Fig. 4). tDCS alone at a WTP threshold of $50,000 per QALY had a 54% probability of being cost-effective, 80% at a $100,000 per QALY threshold.

**Table 4:** Base care costs, quality adjusted life years (QALYs) and incremental cost and QALYs compared to standard care. Costs reported in 2020 Canadian dollars.

Strategy	Mean total cost (95% confidence interval)	Incremental cost	Mean total QALYs (95% confidence interval)	Incremental QALYs	Incremental cost effective ratio
Standard care	$7,393 ($7,125–$7,672)		0.117 (0.116–0.119)		
Transcranial direct current stimulation	$8,066 ($7,861–$8,276)	$673	0.137 (0.136–0.139)	0.020	$33,167
Virtual illusion and transcranial direct current stimulation	$7,547 ($7,395–$7,701)	$155	0.163 (0.162–0.164)	0.046	$3,396

The total cost, QALYs and ICER for each scenario analysis conducted is presented in Table 5. Excluding the productivity costs from the analysis increased ICER values. Expanding the timeframe of the analysis resulted in VI and tDCS and tDCS dominating (lower cost and better QALY) standard care. Changes in utility and cost model inputs resulted in ICERs for VI and tDCS ranging between $1600 and $6100 per QALY. The ICER for tDCS alone was between $14,600 and $33,200.

**Table 5:** Results of scenario sensitivity analyses. Results reported in 2020 Canadian dollars, rounded to nearest tens.

Strategy	Mean total cost (95% confidence interval)	Incremental cost	Mean total QALYs (95% confidence interval)	Incremental QALYs	Incremental cost effective ratio
**Scenario 1: Public Healthcare Payer perspective**					
Standard care	$3,339 ($3,261–$3,425)		0.118 (0.117–0.120)		
Transcranial direct current stimulation	$4,455 ($4,398–$4,509)	$1,116	0.138 (0.137–0.140)	0.020	$56,019
Virtual illusion + transcranial direct current stimulation	$4,719 ($4,676–$4,767)	$1,379	0.163 (0.162–0.164)	0.0448	$30,808
**Time frame**					
**Scenario 2: 1 year**					
Standard care	$22,862 ($20,771–$25,301)		0.448 (0.442–0.455)		
Transcranial direct current stimulation	$21,317 ($19,904–$22,832)	−$1,544	0.521 (0.517–0.526)	0.073	tDCS dominates standard care.
Virtual illusion + transcranial direct current stimulation	$15,940 ($15,271–$16,574)	−$6,922	0.647 (0.643–0.651)	0.198	VI + tDCS dominates standard care.
**Scenario 3: 2 years**					
Standard care	$39,409 ($36,305–$42,899)		0.875 (0.863–0.887)		
Transcranial direct current stimulation	$36,229 ($34,053–$38,614)	−$3,180	1.018 (1.010–1.027)	0.143	tDCS dominates standard care.
Virtual illusion + transcranial direct current stimulation	$26,209 ($25,106–$27,260)	−$13,200	1.279 (1.271–1.287)	0.404	VI + tDCS dominates standard care.
**Utilities**					
**Scenario 4: Study by Dixon and colleagues**					
Standard care	$7,393 ($7,112–$7,672)		0.090 (0.089–0.092)		
Transcranial direct current stimulation	$8,066 ($7,858–$8,270)	$673	0.136 (0.135–0.138)	0.046	$14,602
Virtual illusion + transcranial direct current stimulation	$7,547 ($7,403–$7,699)	$155	0.187 (0.186–0.188)	0.097	$1,600
**Scenario 5: Study by Gordon and colleagues**					
Standard care	$7,393 ($7,112–$7,681)		0.052 (0.051–0.054)		
Transcranial direct current stimulation	$8,066 ($7,862–$8,274)	$673	0.081 (0.08-0–082)	0.029	$23,559
Virtual illusion + transcranial direct current stimulation	$7,547 ($7,402–$7,697)	$155	0.118 (0.118–0.120)	0.066	$2,342
**Costs**					
**Scenario 6: Alternative VR equipment costs**					
Standard care	$7,393 ($7,107–$7,695)		0.117 (0.115–0.119)		
Transcranial direct current stimulation	$8,066 ($7,878–$8,272)	$673	0.137 (0.136–0.139)	0.020	$33,167
Virtual illusion + transcranial direct current stimulation	$7,670 ($7,514–$7,825)	$277	0.163 (0.162–0.164)	0.046	$6,086

Note: QALY, quality-adjusted life years; ICER, incremental cost-utility ratio.

## Discussion

After three months, VI and tDCS therapy and tDCS alone are expected to result in slightly higher cumulative incremental costs and higher incremental QALYs from a societal perspective. Within three months, the initial cost of including VI to tDCS therapy is almost completely offset by reductions in health care costs resulting from improvements in NP severity. Likewise, there are improvements in health-related quality of life as a result of better NP outcomes. By one-year post-treatment, cumulative costs for the two treatments were lower than standard care. VI and tDCS therapy had larger negative incremental cost and QALY improvements compared to tDCS therapy alone. Our results remained relatively robust with alternative model inputs in the eight scenario analyses we carried out. Examining the results from the health care payer perspective there is a little over 50% probability that tDCS therapy is cost effective at a $50,000 per QALY WTP, while there is a 74% probability that VI and tDCS therapy is cost effective. The results observed in our study could not be compared to previous studies due to a lack of economic analyses evaluating treatments for NP in the SCI population and tDCS or VI therapy in general. This represents a large knowledge gap that requires further study.

Several conservative data inputs were used in the base case to bias our results to favor standard care. For example, when adding the final costs associated with being in a particular health state to extend our time horizon to one year, we used costs derived from a study by Hogan *et al.*^50^ for the base case analysis. This reported on costs of a combination of various types of chronic pain, including arthritis, back and neck problems, fibromyalgia, migraine, NP, for each pain severity (mild, moderate, and severe) based on Canadian census data.^50^ Previous studies have demonstrated that NP was more costly than other types of chronic pain,^8,53^ suggesting that our use of annual costs from Hogan *et al.*^50^ was a conservative estimate. Indeed, when we used costs for NP alone derived from a US study^51^ in our scenario analysis, we observed a lower ICER value. However, since healthcare costs vary greatly from the US to Canada due to differences in healthcare systems, we decided to use a conservative estimate for our base case analysis. Additionally, productivity costs were derived from absenteeism data,^46^ but not presenteeism. In our scenario analysis using absenteeism and presenteeism productivity costs from the United States,^51^ we observed a large decrease in the total cost of the intervention resulting in a large decrease in the resulting ICER. Ultimately, our base case included various conservative cost estimates biased towards the comparator, resulting in a more conservative ICER value.

Strengths of our study included addressing uncertainties around parameter estimates through probabilistic sensitivity analysis and various scenario analyses. Additionally, the clinical trial data we used only recruited individuals with NP after SCI who were not responding to drug treatments, reflecting the anticipated population that would receive VI and tDCS therapy in a real-world clinical setting.

Our study had a number of limitations. First, our patient-level data for both the intervention and the comparator were derived from clinical trials with small sample sizes of less than 20 participants,^15,16,31,32^ leading to increased uncertainty around the data inputs used. Data input uncertainty was incorporated in our analysis by assigning distributions with large standard deviations to model variables in our probabilistic model. At a high WTP of $100,000 there is still only a 10% and 20% probability that VI and tDCS and tDCS alone is not cost effective. This signifies that there remains some uncertainty in the cost-effectiveness results. Another limitation is the exclusion of adverse event related costs because the frequency of these events at each NP pain severity level were not available. Clinical studies on VI and tDCS treatment have not observed any differences in adverse outcomes.^15^ Thus, it is not expected that the inclusion of adverse event related cost would change the incremental outcomes. A third limitation was the short time horizon of our study. Although our time horizon was the same as that used in previous studies,^25,28,29^ a three month time horizon may not capture future costs and benefits associated with the VI and tDCS treatment for NP. Due to the short clinical trial follow-up period and lack of long-term studies, it would be difficult to predict the effect of either VI and tDCS therapy or tDCS therapy alone on NP pain intensity over a period exceeding one year. Furthermore, there have been no studies that have followed up on the costs of a NP population over a duration of longer than a year. As such, the cost of NP may be higher or lower in comparison to our extrapolated costs over a one-year time horizon. Further studies are needed to quantify the long-term impact of the intervention on NP pain intensity and costs. Another limitation of our study was exclusion of out-of-pocket and caregiving costs. These costs are expected to increase with increasing levels of pain severity and would result in a more favorable ICER for the intervention.^8,53,54^ Unfortunately, this data is unavailable and should be explored in future economic analyses. Lastly, it should be noted that our economic evaluation may not be generalizable to other non-immersive or immersive virtual reality interventions that use different equipment.^14^

Overall, VI and tDCS therapy appears to be cost-effective at a $50 000 WTP threshold over a three-month time horizon from a Canadian public payer and societal perspective. On the other hand, whether tDCS therapy alone is cost-effective at the same threshold remains uncertain. At longer timeframes, VI and tDCS and tDCS alone are estimated to decrease costs. Comparing the results for VI and tDCS therapy to tDCS alone, the inclusion of VI to tDCS therapy resulted in a lower incremental cost and greater QALY than tDCS alone. As such, VI and tDCS therapy should be considered a viable option for treatment of NP following SCI. However, this recommendation should be treated with caution as more clinical trials with larger samples sizes investigating the long-term effects of VI and tDCS therapy on pain intensity and secondary complications in individuals with NP and SCI are needed. There are limited treatment options for individuals who continue to experience NP despite receiving pharmacological therapies. This lack of treatment alternatives results in a substantial economic burden for health care payers and individuals with NP. Our analysis suggests that based on current clinical evidence, VI and tDCS therapy may be a cost-effective option for individuals with SCI experiencing NP resistant to standard care.

## Conclusion

Overall, we found that virtual illusion and tDCS combination therapy was cost-effective at a $50,000 WTP threshold over a one-year time horizon from a Canadian public payer and societal perspective. As such, public funding for virtual illusion and tDCS combination therapy should be considered for the treatment NP following SCI. However, this recommendation should be treated with caution as more clinical trials with larger samples sizes investigating the long-term effects of virtual illusion and tDCS combination therapy on pain intensity and secondary complications in individuals with NP and SCI are needed.

## Appendices

**Table A1:** Healthcare utilization probabilities, and unit cost (2020 Canadian dollars) model inputs.

Parameter	Model inputs	Distribution type	Reference
**Healthcare utilization probabilities, mean (standard deviation)**			
Probability of physician visit – mild	0.24 (0.1)	Beta	Tarride *et al.*^29^
Probability of physician visit – moderate	0.45 (0.1)	Beta	Tarride *et al.*^29^
Probability of physician visit – severe	0.61 (0.1)	Beta	Tarride *et al.*^29^
Probability of specialist referral – mild	0.05 (0.1)	Beta	Tarride *et al.*^29^
Probability of specialist referral – moderate	0.1 (0.1)	Beta	Tarride *et al.*^29^
Probability of specialist referral – severe	0.20 (0.1)	Beta	Tarride *et al.*^29^
Probability of CT scan	0.09	–	Tarride *et al.*^29^
Probability of MRI	0.1	–	Tarride *et al.*^29^
Probability of nerve conduction study	0.31	–	Tarride *et al.*^29^
Probability of quantitative sensory test	0.07	–	Tarride *et al.*^29^
Probability of doppler sonography	0.045	–	Tarride *et al.*^29^
Probability of electromyography	0.23	–	Tarride *et al.*^29^
Probability of glycosylation test	0.015	–	Tarride *et al.*^29^
Probability of creatine test	0.015	–	Tarride *et al.*^29^
Probability of complete blood count test	0.02	–	Tarride *et al.*^29^
Probability of drug infiltration test	0.13	–	Tarride *et al.*^29^
Probability of nerve block test	0.185	–	Tarride *et al.*^29^
Probability of transcutaneous electrical nerve stimulation	0.155	–	Tarride *et al.*^29^
Probability of implementation of spinal stimulator	0.01	–	Tarride *et al.*^29^
**Healthcare utilization costs, mean**			
Cost per physician visit	84.45	–	Ontario Physician Schedule of Benefits (A003)^42^
Cost per specialist consult	87.60	–	CIHI^56^
Cost per CT scan ordered^a^	399.69	–	Ontario Physician Schedule of Benefits (X128), Tarride *et al.*^29,42^
Cost per MRI ordered^a^	1,363.07	–	Ontario Physician Schedule of Benefits (X496), Tarride *et al.*^29,42^
Cost per nerve conduction study ordered^a^	605.24	–	Ontario Physician Schedule of Benefits (G455+G456), Tarride *et al.*^29,42^
Cost per quantitative sensory test ordered^a^	657.59	–	Ontario Physician Schedule of Benefits (G466+G457), Tarride *et al.*^29,42^
Cost per doppler sonography ordered^a^	335.87	–	Ontario Physician Schedule of Benefits (J290), Tarride *et al.*^29,42^
Cost per electromyography ordered^a^	814.01	–	Ontario Physician Schedule of Benefits (G455+G456), Tarride *et al.*^29,42^
Cost per glycosylation test ordered^a^	18.01	–	Ontario Schedule of Benefits for Laboratory Services (L093+L700), Tarride *et al.*^43,46^
Cost per creatine test ordered^a^	24.2	–	Ontario Schedule of Benefits for Laboratory Services (L065+L700), Tarride *et al.*^43,46^
Cost per complete blood count test ordered^a^	14.74	–	Ontario Schedule of Benefits for Laboratory Services (L393+L700), Tarride *et al.*^43,46^
Cost per drug infiltration test ordered^a^	180	–	Ontario Physician Schedule of Benefits (G245), Tarride *et al.*^29,42^
Cost per nerve block test ordered^a^	395.74	–	Ontario Physician Schedule of Benefits (G231), Tarride *et al.*^29,42^
Cost per transcutaneous electrical nerve stimulation ordered^a^	93.87	–	Tarride *et al.*^29^
Cost per implementation of spinal stimulator ordered^a^	25,828.59	–	Ontario Physician Schedule of Benefits (Z823), Tarride *et al.*^29,42^

^a^ In the model, the cost of a particular diagnostic test was multiplied by the probability of a physician visit and specialist referral.

**Table A2:** Productivity cost (2020 Canadian dollars) model inputs.

Parameter	Model inputs	Distribution type	Reference
Productivity costs per patient per month, mean (standard deviation)			
Missed number of paid work hours per month – mild^a^	1 (4)	Gamma	Tarride *et al.*^46^
Missed number of paid work hours per month – moderate^a^	4 (13)	Gamma	Tarride *et al.*^46^
Missed number of paid work hours per month – severe^a^	6 (26)	Gamma	Tarride *et al.*^46^
Missed number of unpaid work hours per month – mild^b^	11 (26)	Gamma	Tarride *et al.*^46^
Missed number of unpaid work hours per month – moderate^b^	52 (67)	Gamma	Tarride *et al.*^46^
Missed number of unpaid work hours per month – severe^b^	72 (77)	Gamma	Tarride *et al.*^46^
Average hourly wage	29.72	–	Statistics Canada^47^
Minimum hourly wage	14	–	Ontario Ministry of Labour^48^

^a^ Cost of missed paid work was modeled by multiplying the number of hours of missed paid work by the average hourly wage in Canada as per Tarride *et al.*^46^

^b^ Cost of missed unpaid work was modeled by multiplying the number of hours of missed unpaid work by the minimum hourly wage in Ontario as per Tarride *et al.*^46^

**Table A3:** Long-term direct healthcare and productivity cost (2020 Canadian dollars) model inputs for one and two-year scenario analyses.

Parameter	Model inputs	Distribution type	Reference
Final costs per patient of remaining in a pain state for one year, mean (standard deviation)			
Direct healthcare costs – mild	652.94 (5,553)	Gamma	Hogan *et al.*^50^
Direct healthcare costs – moderate	1,787.97 (4,838)	Gamma	Hogan *et al.*^50^
Direct healthcare costs – severe	4,309.41 (27,652)	Gamma	Hogan *et al.*^50^
Productivity costs – mild	2,310.60 (10,449)	Gamma	Tarride *et al.*^46^
Productivity costs – moderate	10,221.12 (15,752)	Gamma	Tarride *et al.*^46^
Productivity costs – severe	14,159.52 (22,098)	Gamma	Tarride *et al.*^46^
